# Evasion of NK cell immune surveillance *via* the vimentin-mediated cytoskeleton remodeling

**DOI:** 10.3389/fimmu.2022.883178

**Published:** 2022-08-11

**Authors:** Jei-Ming Peng, Ching-Feng Chiu, Jai-Hong Cheng, Hui-Ying Liu, Yin-Lun Chang, Jia-Wun Luo, Yu-Ting Weng, Hao-Lun Luo

**Affiliations:** ^1^ Institute for Translational Research in Biomedicine, Kaohsiung Chang Gung Memorial Hospital, Kaohsiung, Taiwan; ^2^ Graduate Institute of Metabolism and Obesity Sciences, Taipei Medical University, Taipei, Taiwan; ^3^ Center for Shockwave Medicine and Tissue Engineering, Medical Research, Kaohsiung Chang Gung Memorial Hospital and Chang Gung University College of Medicine, Kaohsiung, Taiwan; ^4^ Department of Leisure and Sports Management, Cheng Shiu University, Kaohsiung, Taiwan; ^5^ Department of Urology, Kaohsiung Chang Gung Memorial Hospital and Chang Gung University College of Medicine, Kaohsiung, Taiwan

**Keywords:** natural killer cell, immune surveillance, vimentin, intracellular cytoskeleton remodeling, immunotherapy

## Abstract

Cancer immunotherapy uses the immune system to achieve therapeutic effects; however, its effect is still limited. Therefore, in addition to immune checkpoint-based treatment, the development of other strategies that can inhibit cancer cells from resisting immune cytotoxicity is important. There are currently few studies on the mechanism of tumors using cytoskeletal proteins reorganization to participate in immune escape. In this study, we identified cancer cell lines that were sensitive or resistant to natural killer cells in urothelial and lung cancer using the natural killer cell sensitivity assay. We found that immunoresistant cancer cells avoid natural killer cell-mediated cytotoxicity by upregulation of vimentin and remodeling of actin cytoskeleton. Immunofluorescence staining showed that immune cells promoted the formation of actin filaments at the immune synapse, which was not found in immunosensitive cancer cells. Pretreatment of the actin polymerization inhibitors latrunculin B increased the cytotoxicity of natural killer cells, suggesting that cytoskeleton remodeling plays a role in resisting immune cell attack. In addition, silencing of vimentin with shRNA potentiated the cytotoxicity of natural killer cells. Interestingly, the upregulation and extension of vimentin was found in tumor islands of upper tract urothelial carcinoma infiltrated by natural killer cells. Conversely, tumors without natural killer cell invasion showed less vimentin signal. The expression level of vimentin was highly correlated with natural killer cell infiltration. In summary, we found that when immune cells attack cancer cells, the cancer cells resist immune cytotoxicity through upregulated vimentin and actin reorganization. In addition, this immune resistance mechanism was also found in patient tumors, indicating the possibility that they can be applied to evaluate the immune response in clinical diagnosis.

## Introduction

For the upper tract urothelial carcinoma (UTUC), the incidence was less than 10% in the United Sates, whereas it was very high in Taiwan, being up to 30% of urothelial carcinomas (1). UTUC and bladder cancer belong to the same urothelial cell type, but their clinical prognoses are very different. Tumors invade the muscular layer in 60% of UTUC while invasion is about 10–20% in bladder cancers. Although most tumors can be completely removed, a high percentage of patients die from disease progression and distant metastasis (2). UTUC is often accompanied by renal insufficiency, which makes it difficult for the patient to receive an adequate dose of chemotherapy (3). Therefore, invasive or metastatic UTUC has a very poor clinical prognosis. At present, effective biomarkers are still quite limited, and it is necessary to identify markers to predict tumors and prognoses. There is an urgent need to develop more effective treatment strategies and improve prognosis with chemotherapy (4).

The increase in cancer mortality affects human health and social development, and finding safe and effective treatments is a challenge for cancer researchers worldwide. Traditional cancer treatments, such as surgical, radiotherapy and chemotherapy, do not ensure patient outcomes. In recent years, the development of gene targeted therapy has extended the survival of tumor patients, but the recurrence after treatment is still high. Immunotherapy, which aims to activate the patient’s immune system to kill cancer cells, has been studied most at immune checkpoint inhibitors, while others include chimeric antigen receptor T cell therapy (CAR‐T). Tumor cells can inhibit the function of natural killer cells (NK) and cytotoxic T lymphocytes (CTLs) through cytokines, chemokines and metabolites, thus helping tumor cells escape recognition and attack by the immune system (5). Among these immune cells, NK cells play a key role in the first-line immune response and are characterized by rapid response. The main approaches to NK cell-based cancer treatment include cytokines, antibodies, and adoptive transfer of NK cells by increasing the persistence, activation, number, or targeting of tumor cells (6)

Recent literature reveals that treatment of TGF-β blocking agent can enhance immune cell response and infiltration (7). Upregulation of TGF-β1 mediated vimentin is one of the key hallmarks of epithelial–mesenchymal transition (EMT) progression (8). Vimentin is an intermediate filament protein and a member of cytoskeletal proteins. TGF-β1 drives vimentin activation, which plays a key role in the cytoskeleton remodeling and mobility during EMT (9). Recent studies have found that vimentin can be detected in many types of cancers, including lung, colon, cervical and prostatic cancers, and the expression of vimentin is highly correlated with extent of these tumors (8, 10–12). vimentin is originally expressed in mesenchymal cells, such as fibroblasts, chondrocytes, macrophages and endothelial cells, rather than in epithelial cells (13). The determination of vimentin was thought to be as a predictive factor of poor prognosis in patients with gastric cancer (14), but this result is still controversial in other cancers (15). Thus, exploring the mechanism and the role of TGF-β or vimentin mediated signaling in the immune cell response can provide a clearer definition in patient samples.

Mechanotransduction in cancer cells contain variable processes of physical structure rearrangement that force chemical stimulation and cause signal transduction of cell function (16, 17). Our results show that vimentin upregulation and cytoskeleton remodeling are the novel tumor suppression strategy for enhancing immune cell-mediated cytotoxicity. Both silencing of vimentin and inhibiting the formation of actin filaments increased cytotoxicity of NK cells. Excitingly, these results are even more obvious in tumor tissue from patients with UTUC. It is currently unclear how vimentin and cytoskeleton remodeling affect the immune cell response in the primary and invasive tumors. Here, we uncover a novel strategy for improving clinical diagnosis by regulating vimentin and cytoskeleton remodeling to modulate the immune response of cancer cells.

## Materials and methods

### Cell lines and materials

Human cells, HT24 (bladder cancer, HTB-4, ATCC), J82 (bladder cancer, HTB-1, ATCC), BFTC909 (colorectal cancer, 60069, BCRC), H292 (lung cancer, CRL-1848, ATCC), A549 (lung cancer, CCL-185, ATCC) and NK-92MI (lymphoma, CRL-2408, ATCC) were obtained from the American Type Culture Collection (ATCC) or Bioresource Collection and Research Center (BCRC). Cells were cultured in DMEM medium (Thermo Fisher Scientific, Waltham, MA, USA) containing 10% (volume/volume; v/v) fetal bovine serum (FBS), sodium bicarbonate and 1% (v/v) antibiotic-antimycotic (Thermo Fisher Scientific, Waltham, MA, USA) at 37°C in an incubator with a humidified atmosphere of 5% CO2. NK-92MI cells were cultured in alpha minimum essential medium (Thermo Fisher Scientific) supplemented with 12.5% fetal bovine serum (FBS, Sigma-Aldrich, St. Louis MO, USA), 2 mM L-glutamine (Thermo Fisher Scientific), 1.5 g/L sodium bicarbonate (Thermo Fisher Scientific), 0.2 mM inositol; (I7508, Sigma-Aldrich), 0.1 mM 2-mercaptoethanol; (M6250, Sigma-Aldrich), 0.02 mM folic acid; (F8758, Sigma-Aldrich), 12.5% horse serum. Latrunculin B (L5288, Sigma-Aldrich). Antibodies, CD161/NK1.1 was from Novus Biologicals (NB100-77528SS, Littleton, CO, USA), vimentin was from abcam (ab92547, Cambridge, UK). Phalloidin staining reagent was from Abcam (ab112125, Cambridge, UK).

### Natural killer cell cytotoxicity assay

Cells were seeded at 2 x 10^4^ or 4 x 10^4^ cells per well in a 96-well plate (Jet Biofil, Guangzhou, China). Next day, NK-92MI cells were added into well in a serial dilution for 24 or 48 h (dilution 1:0.25, 1:0.5, 1:1, 1:3, 1:6 in culture media, #356234, Corning, Bedford, MA, USA). The fixed cells were stained with crystal violet (0.1 mg/mL) for 2 h, and the images were photographed under an inverted microscope (IX51, Olympus, Japan). The crystal violet staining cells were quantitatively analyzed and modified according to the study of Cvetannova et al. (18). The crystal violet-stained cells were then dissolved in 250 μL 20% acetic acid. Absorbance (O.D. 595 nm) was measured using an ELISA reader (Varioskan LUX Multimode Microplate Reader, Thermo Fisher Scientific, USA). The plots of NK cell cytotoxicity were analyzed using GraphPad Prism 8 software (GraphPad, San Diego, CA, USA).

### shRNA and lentivirus infection

RNA was knocked down using lentivirus containing the shRNA for empty vector (shEV) or vimentin (shVimentin), virus was obtained from National RNAi Core in Taiwan. Cells were seeded at 1 x 10^5^ cells per well in a 12-well plate and infected with shEV and shVimentin with MOI 2 for 48 h prior to puromycin selection. The shRNA targeting sequences of vimentin were shVimentin#1: GCTAACTACCAAGACACTATT and shVimentin#2: GCAGGATGAGATTCAGAATAT. PCR was performed using PFU Turbo polymerase (Agilent, Santa Clara, CA, USA) according to the manufacturer’s instructions.

### Quantitative real-time PCR

RNA was isolated using TRIzol reagent (Thermo Fisher Scientific, Waltham, MA, USA) and cDNA was synthesized using SuperScript™ III Reverse Transcriptase (Thermo Fisher Scientific). qRT-PCR was performed using SYBR Green PCR Master Mix (Thermo Fisher Scientific, Waltham, MA, USA) and ABI StepOnePlus sequence detection system (Thermo Fisher Scientific, Waltham, MA, USA). The real-time PCR primers were as follows: Vimentin forward, 5′-GACGCCATCAACACCGAGTT-3′; Vimentin reverse primer, 5′-CTTTGTCGTTGGTTAGCTGGT-3′; GAPDH forward, 5′-TGAAGGTCGGAGTCAACGGATT-3′; GAPDH reverse primer, 5′-CCTGGAAGATGGTGATGGGATT-3′.

### Immunofluorescence staining

The cancer cell lines (2 x 10^4^ cells) were seeded on coverslips in a 12-well plate overnight. The next day, the NK-92MI cells were stained with CellTracker Red CMTPX dye (C34552, Thermo Fisher Scientific) for 30 min and washed twice with PBS. Cancer cells were treated with CMTPX-stained NK-92MI cells for 2, 6, and 24 h (dilution, 1:0.5). Cells were fixed in 4% paraformaldehyde for 20 min and parametrization was performed in 0.1% Triton X-100 for 15 min. Blocking buffer (1% BSA, 0.1 M glycine) was added for 30 min and then reacted with first antibodies (1:200) in blocking buffer overnight. The samples were washed with PBST (0.1% Tween-20) three times and then secondary antibodies conjugated with Alexa Fluor 488 or Alexa Fluor 594 (1:200) in blocking buffer were added for 2 h. Samples were stained with DAPI and mounted with Prolong Gold antifade reagent (Thermo Fisher Scientific, Waltham, MA, USA). The samples were stored at 4°C in the dark. Fluorescent cells were observed using a confocal laser scanning microscope (FluoView FV10i, Olympus, Tokyo, Japan). Ten fields for each experiment were examined at 100-fold magnification and up to 630-fold magnification to obtain detailed images. Quantification of β-tubulin and vimentin was performed after the cells were selected, and the intensity of the fluorescent protein was analyzed with and without NK cell interaction. Quantitative analysis of actin responses in front regions of the immune synapse bound to NK cells, marked with dotted rectangles (F), and unbound regions at the rear, marked with dotted rectangles (R), was performed. Fluorescence intensity (unit) = F-R in the immune synapse, showing the level of F-actin aggregation. Fluorescence intensity and quantification were analyzed using the FV10-ASW software (version 4.0) and ImageJ software (version 1.8). For analysis of immunofluorescence staining of tumor sections, a Vectra Polaris Automated Quantitative Pathology Imaging System (PerkinElmer, Boston, MA, USA) was used to scan the slides. Fluorescence intensity and quantification were analyzed using inForm software (version 2.3, PerkinElmer).

### Immunohistochemistry staining

Tumors were fixed in 10% formalin overnight and embedded in paraffin for sectioning. The sections were cut at 4-5 µm. Tumor sections were deparaffinized in xylene and antigen retrieval reagent was added for 30 min (Agilent Dako, Santa clara, CA, USA). Tissue sections were added blocking buffer (1% BSA, 0.1% Tween-20) for 30 min and then reacted with first antibodies (1:200) in blocking buffer overnight. The samples were washed with PBST for three times and then added secondary antibodies-conjugated with horseradish peroxidase (HRP) (1:200) in blocking buffer for 2 h. Samples were counterstained with hematoxylin and stained with 3,3’-Diaminobenzidine (DAB). For analysis of tumor sections, a Vectra Polaris Automated Quantitative Pathology Imaging System (PerkinElmer, Boston, MA, USA) was used to scan the slides. Intensity and quantification were analyzed using inForm software (version 2.3, PerkinElmer).

### Western blot assay

Cells were washed in ice-cold PBS and lysed in a RIPA buffer containing 50 mM Tris-HCl pH 8.0, 120 mM NaCl, 0.5% NP-40, 0.25% Na deoxycholate, 1 mM DTT, 1 mM PMSF, 1 mM EDTA, 1 mM NAF, 1 mM Na_3_VO_4_, 2 mg/mL aprotinin, and 2 mg/ml leupeptin. Cellular debris were removed by centrifugation at 13 krpm at 4°C for 15 min. The protein lysates were quantified by BCA assay (Thermo Fisher Scientific, Waltham, MA, USA) and equal amounts of protein (20 µg) was resolved on SDS polyacrylamide gels, transferred to PVDF membranes (Schleicher & Schuell, Dassel, Germany), and then incubated with primary antibodies overnight. After reaction with HRP-conjugated secondary antibody (1:2000 dilution; Cell Signaling Technology, Beverly, MA, USA) for 2 h, each membrane was scanned using a UVP ChemStudio PLUS instrument (UVP Inc., Upland, CA, USA) and analyzed with the ImageJ software (version 1.8).

### Statistical analysis

Unless otherwise stated, all experiments were conducted at least three times. Data from the NK cytotoxicity assay, real-time qPCR, immunohistochemistry staining in this study was expressed as mean ± s.d. Statistical significance between different experimental groups was analyzed using the Student’s t-test (two-tailed), 1-way with Dunnett’s multiple comparisons test or 2-way ANOVA with Tukey’s multiple comparisons test. P values less than 0.05 were considered significant. Statistical analyses were performed using GraphPad Prism 8 (GraphPad software, San Diego, CA, USA).

## Results

### Identifying cancer cell groups that were sensitive or resistant to immune cells

**Figure 1:**
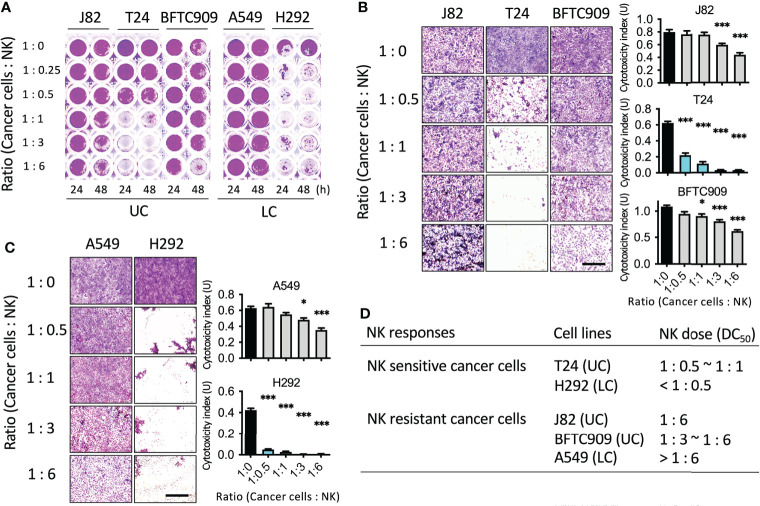
Identification of the cancer cell lines susceptible or resistant to NK cell-mediated cell death.** (A)** NK cytotoxicity assay using cancer cells from urothelial cancer (UC) and lung cancer (LC). Cells were seeded in the 96-well plate and incubated with NK cells for 24 or 48 h. **(B)** J82, T24 and BFTC909 cells were co-cultured with NK cells for 48 h and stained with crystal violet. The stained cells were observed by microscope. **(C)** A549 and H292 cells were co-cultured with NK cells for 48 h and stained with crystal violet. The stained cells were observed by microscope. **(D)** The NK sensitive or resistant cancer cells and the dose of NK ratio are listed. Scale bars, 1 mm. *P < 0.05; ***P < 0.001. Data are presented as mean ± s.d. (one-way ANOVA with Dunnett’s multiple comparisons test) and experiments were repeated three times (n = 3).

Currently, few studies have investigated the mechanism of the involvement of cytoskeleton-associated proteins of cancer cells in immune cell tolerance (19, 20). First, a NK cell cytotoxicity assay was performed to explore the mechanism by which cancer cells resist cell death caused by NK cells. The purpose of this analysis was to identify the sensitivity of cancer cells to the cytotoxicity of immune cells. Cancer cells used in the analysis were urothelial cancer cells (J82, T24, and BFTC909). Since the immune response regulated by NK cells had been widely studied in lung cancer, the lung cancer cells (H292 and A549) were also studied. Cancer cells were seeded into 96-well plates and incubated for 24 h. Next day, different proportions of NK cells were added (NK-92MI). The ratios of cancer cells to NK cells were 1:0.25, 1:0.5, 1:1, 1:3, and 1:6. The cancer cells and immune cells were co-incubated for 24 or 48 h and then fixed and stained with crystal violet dye (**Figure 1A**). To quantify the number of cells, acetic acid was used to dissolve the crystal violet dye and absorbance was measured. T24 cells were found to be more sensitive to NK cells than the other two cell lines. Over 90% of the cells died at an NK cell ratio of 1:1. In contrast, J82 and BFTC909 cell lines were resistant to NK cells, with approximately 20% of cell death occurring after incubation with NK cells at a ratio of 1:3 for 48 h. The crystal violet-stained cells in **Figure 1A** were observed using a microscope to observe the morphology of cancer cells after treatment with NK cells (**Figure 1B**). The shape of the T24 cells changed from round to elongated at a ratio of 1:1 (**Figure 1B**). Only some J82 and BFTC909 cells appeared to shrink at a ratio of 1:6, while the remaining cells showed normal morphology. The crystal violet-stained cells from the plate in **Figure 1A** were quantitatively analyzed and modified according to the study of Cvetanova et al. (18). The fixed cells were dissolved in 20% acetic acid, and the absorbance at 595 nm was measured using an ELISA reader (**Figure 1B**, right panel). Statistical quantification of cells revealed that when treated with NK cells at a ratio of 1:3, the rate of T24 cell death was 95.8%, whereas that of J82 and BFTC909 cells were 22.4% and 15.4%, respectively (**Figure 1B**). This result showed that T24 cells were sensitive to NK cells, while J82 and BFTC909 cells were resistant to cancer cells.

We then investigated the cytotoxicity of NK cells on lung cancer cells. After adding NK cells at different ratios, cancer and immune cells were incubated for 24 and 48 h, respectively, and the cells were then fixed and stained with crystal violet dye (**Figure 1C**). H292 cells were found to be more sensitive to NK cells than A549 cells. Over 90% of cells died when incubated with NK cells at a ratio of 1:0.5. In contrast, A549 cells were resistant to NK cells, with approximately 20% cell death occurring in the presence of NK cells at a ratio of 1:3, indicating that A549 cells had low sensitivity to NK cells. The morphology of cancer cells after treatment with NK cells was observed with a microscope, and H292 cells were found with round shape in the presence of NK cells a ratio of 1:0.5 (**Figure 1C**). However, at a ratio of 1:6, only a few A549 cells shrank and the rest of the cells had normal morphology. Statistical quantification of the cells showed that when treated with NK cells at a ratio of 1:3, the rate of H292 cell death was 96.2%, while that of A549 cells was 23.6% (**Figure 1C**). This result showed that H292 cells were immunosensitive to NK cells, while A549 were resistant to cancer cells. To summarize, these cells were classified as sensitive or resistant urothelial or lung cancer cells based on their sensitivity to NK cells (**Figure 1D**). The immunosensitive cancer cells were T24 and H292, and the immunoresistant cancer cells were J82, BFTC909, and A549.

### Accumulation of actin filaments was formed at the junction of immune cells and resistant cancer cells

After categorizing the immune sensitive and resistant cancer cells, we then investigated whether they responded differently when attacked by NK cells. In recent years, immunosuppression-related research has mostly focused on immune checkpoint proteins, PD-1 and PD-L1 (21). Although both BFTC909 and A549 were highly resistant to NK cells, A549 was known to express lower levels of PD-L1. This shows that there were other reasons for the immunoresistance of cancer cells. Mariathasan et al. and Tauriello et al. found that the use of TGF-β1-blocking antibody significantly promoted immune cell invasion (7, 22). TGF-β1 has been found to trigger many deteriorating reactions in cancer cells. Most of these reactions result in the reorganization of cytoskeletal proteins and fibrin. Therefore, we further explored whether cytoskeletal proteins of cancer cells affected immune cell attack. The three most important cytoskeletal proteins, namely β-tubulin, vimentin, and actin filaments were screened to observe any significant changes due to immune cell attack. The immunoresistant cancer cell line BFTC909 in **Figure 1** was used to detect changes in these cytoskeletal proteins and fibrin. The experimental design involved co-culture of the resistant cancer cells and NK cells for 6 h (the ratio of cancer cells to NK cells was 1:0.5), followed by fixing of the cells for immunofluorescence staining.

**Figure 2:**
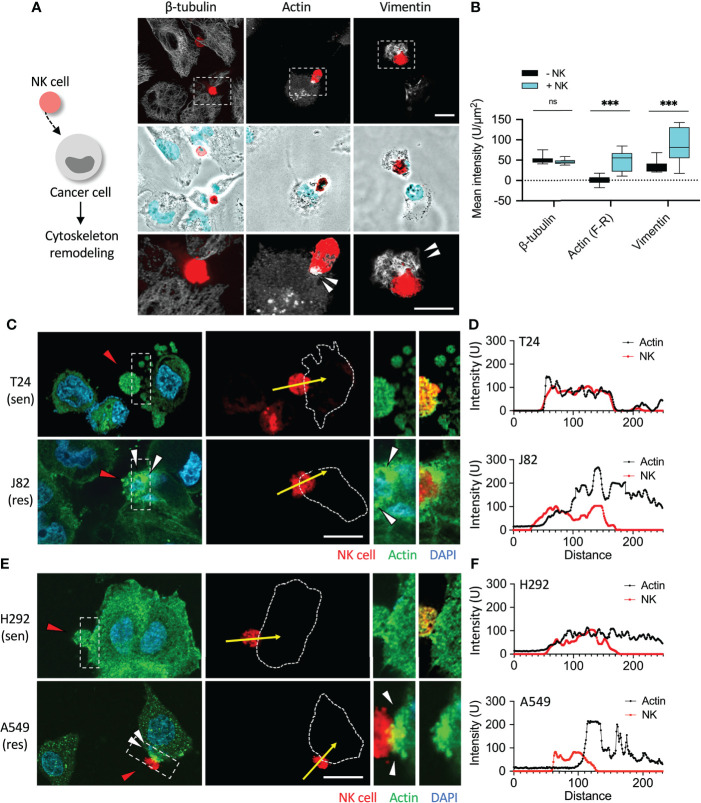
Induction of cytoskeleton remodeling by NK cells. **(A, B)** Comparison of the expression of β-tubulin, vimentin and actin filaments in NK cell-mediated cell lysis. BFTC909 cells were incubated with NK cells for 6 h and the expression β-tubulin, vimentin, and actin filaments was detected by immunofluorescence assays. **(C)** T24 or J82 cells were co-cultured with NK cells for 6 h and stained with a β-actin antibody. The stained cells were observed using a fluorescence microscope. **(E)** H292 and A549 cells were co-cultured with NK cells for 6 h and stained with a β-actin antibody. The stained cells were observed using a fluorescence microscope. **(D, F)** The intensity of prominent fibrous actin near the immunologic synapse was measured and quantified by ImageJ. ns, not significant; ***P < 0.001. Data are presented as means ± s.d. (two-way ANOVA with Sidak's multiple comparisons test). Red arrowhead, NK cell; white arrowhead, actin aggregation. Scale bars, 20 μm. Experiments were repeated at three times (n = 3).

The staining results for β-tubulin, vimentin, and actin filaments showed that NK cells did not cause changes to β-tubulin until 6 h. It was unexpectedly observed in the staining of the other two cytoskeletal proteins that NK cells significantly caused the upregulation of vimentin and actin response (**Figure 2A**). Quantification of β-tubulin and vimentin was performed after the cells were selected, and the intensity of the fluorescent protein was analyzed with and without NK cell interaction. Quantitative analysis of actin responses was performed as described by Al Absi et al. (19). Front regions of the immune synapse bound to NK cells are marked with dotted rectangles (F), and unbound regions at the rear are marked with dotted rectangles (R). Mean fluorescence intensity (unit/µm^2^) = F-R in the immune synapse, showing the level of F-actin aggregation (**Supplementary Figure 1**). After 6 h of NK cell treatment, the expression of β-tubulin, vimentin, and actin filaments was quantified. We performed the quantification of fluorescence intensity in 10 fields (2–3 cells per field) after cells were selected and analyzed, and the results showed that after adding NK cells, the mean intensity of β-tubulin was NK-:NK+ = 50.75:45.48 (P = not significant, ns), mean intensity of actin response was NK-:NK+ = 0.05:47.21 (P < 0.001), and the mean intensity of vimentin was NK-:NK+ = 33.69:83.87 (P < 0.001) (**Figure 2B**). The expression of the fluorescence signal was obvious at 6 h after treatment with NK cells, indicating that vimentin and actin filaments of cancer cells may reflect NK cell attack.

We wanted to further compare whether actin filaments responded differently in sensitive and resistant cancer cells when attacked by NK cells. First, the urothelial cancer cells T24 and J82 were co-cultured with NK cells for 6 h, and no change was observed in actin filaments of T24 cells (**Figure 2C**, and **Supplementary Figure S2A**). However, 6 h after addition of NK cells to the resistant cell line J82, aggregations of actin filaments were clearly observed following the contact between cancer cells and immune cells (**Figure 2C** and **Supplementary Figure S2B**). Quantitative analysis showed that the expression of actin filaments at the immune synapses was high in J82 cells and low in T24 cells (**Figure 2D**).

We then investigated whether actin filaments of sensitive and resistant cancer cells responded differently upon attack by NK cells in lung cancer cells H292 and A549. After co-incubation with NK cells for 6 h, no change in actin filaments was observed in H292 cells (**Figure 2E**, and **Supplementary Figure S2C**). As expected, 6 h after adding NK cells to A549 cells (resistant cells), accumulation of actin filaments was clearly detected (**Figure 2E** and **Supplementary Figure S2D**). We concluded that cancer cells with high immunoresistance produced and aggregated of actin filaments in response to immune cell attack (**Figure 2F**).

**Figure 3:**
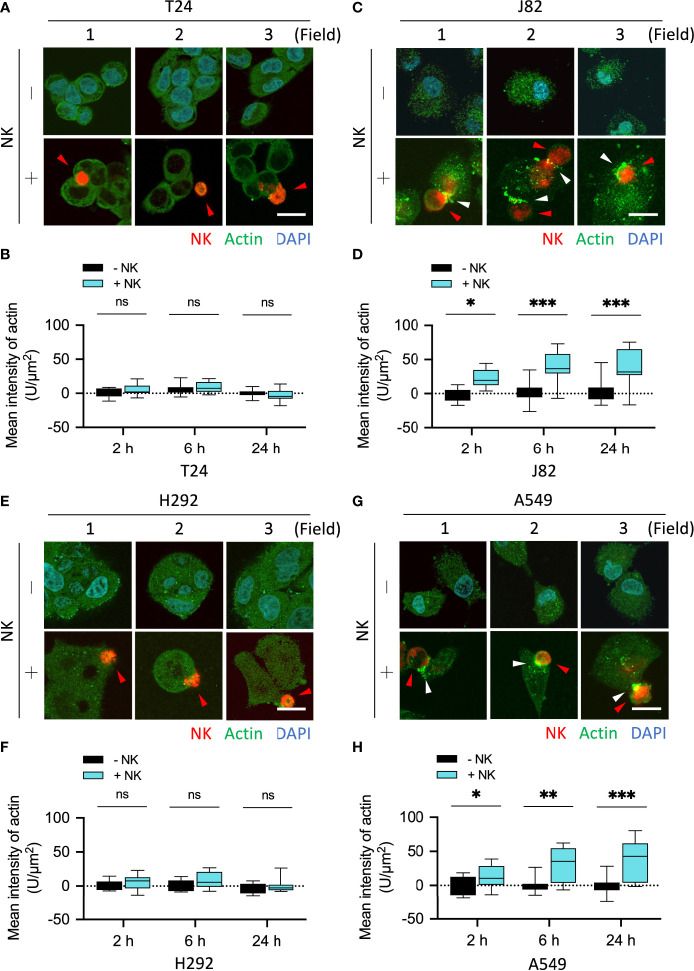
NK cells induced actin responses at the immune synapses in the immune-resistant cancer cells. **(A, C)** Comparison of the expression of actin filaments in NK cell-interacted cancer cells. T24 and J82 cells were incubated with NK cells for 2, 6 and 24 h, and the expression of actin filaments was detected using immunofluorescence assays. **(B, D)** The intensity of actin’s response at the immune synapses was measured and quantified by ImageJ. **(E, G)** H292 and A549 cells were incubated with NK cells for 2, 6 and 24 h, followed by the detection of actin filaments expression. **(F, H)** Quantification of the intensity of actin response at the immune synapses using ImageJ. ns, not significant; *P < 0.05; **P < 0.01; ***P < 0.001. Data are presented as mean ± s.d. (two-way ANOVA with Sidak's multiple comparisons test). Red arrowhead, NK cell; white arrowhead, actin filaments. Scale bars, 20 µm. Data derived from 10 fields, each including 2–3 cells.

To further confirm the effect of NK cells on the organization and expression of cytoskeletal proteins after binding to cancer cells, we co-cultured NK cells with cancer cells and performed immunofluorescence staining in a time-dependent manner. The fluorescence was quantified in 10 fields (2–3 cells each field). The mean fluorescence intensity (F-R) of the actin response at the immune synapse in T24 cells co-cultured with NK cells for 2, 6, and 24 h were NK-:NK+ = 0.01:5.56, 5.96:8.72, and 0.25:-2.53, respectively (all P = ns) (**Figures 3A, B**). The mean fluorescence intensity (F-R) of the actin response at the immune synapse in J82 cells after 2, 6, and 24 h with NK cell co-culture was NK-:NK+ = -1.34:22.12, 1.87:39.56, and 3.84:37.36, respectively (P < 0.05, < 0.001, and < 0.01, respectively) (**Figures 3C, D**). In J82 cancer cells, the mean intensity of actin response was higher after adding NK cells at 6 and 24 h than at 2 h (2, 6 and 24 h; 22.12, 39.56, and 37.36, respectively) (**Figure 3D**).

We performed the same experiment on lung cancer cells and quantified proteins in 10 fields. The mean fluorescence intensity (F-R) of the actin response at the immune synapse in H292 cells co-cultured with NK cells for 2, 6, and 24 h was NK-:NK+ = 1.23:5.55, -1.13:8.85, and -3.38:0.1, respectively (all P = ns) (**Figures 3E, F**). The mean fluorescence intensity (F-R) of the actin response at the immune synapse in A549 cells after 2, 6, and 24 h with NK cell co-culture was NK-:NK+ = -0.54:12.52, -0.36:30.01, and -0.68:37.55, respectively (P = ns, < 0.01, and < 0.001, respectively) (**Figures 3G, H**). Since vimentin can directly interact with actin filaments through its C-terminal tail (23, 24), and mutually affect actin stress fiber assembly and actin-dependent processes such as cell adhesion and migration (9, 25–29), we subsequently explored whether vimentin participates in the resistance response against immune cells.

### Attack by NK cells upregulated vimentin

**Figure 4:**
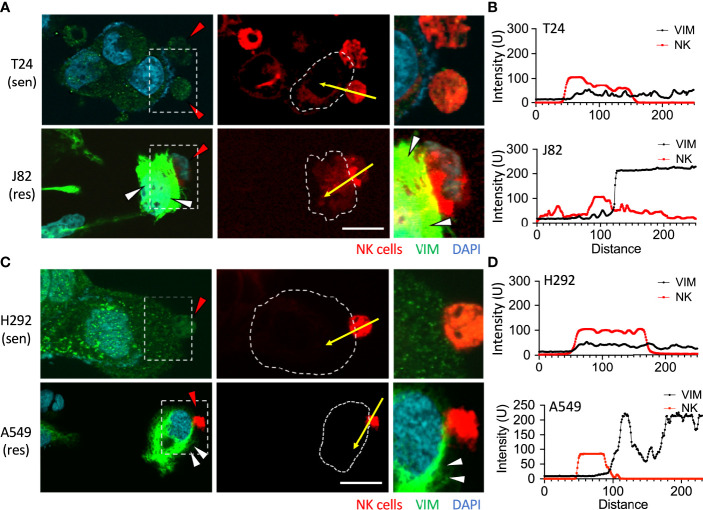
Upregulation of vimentin upon NK cell interaction. **(A)** T24 or J82 cells were co-cultured with NK cells for 6 h and stained with a vimentin antibody. The stained cells were observed using a fluorescence microscope. **(C)** H292 and A549 cells were co-cultured with NK cells for 6 h and stained with a vimentin antibody. The stained cells were observed using a fluorescence microscope. **(B, D)** The intensity of upregulated vimentin in the cancer cells was measured and quantified by ImageJ. Red arrowhead, NK cell; white arrowhead, vimentin upregulation. Scale bars, 20 μm. Experiments were repeated at three times (n = 3).

Jiu et al. demonstrated that one of the functions of vimentin was to facilitate the aggregation and localization of actin. A lack of vimentin disables the cancer cells to promote actin polymerization (25). The results in **Figure 2A** confirmed that the expression of vimentin was significantly increased after NK cell attack. Therefore, a further comparison of the response of vimentin was made between sensitive and resistant cancer cells when attacked by NK cells. Urothelial cancer cells T24 and J82 were used, and after co-cultured with NK cells for 6 h, no change was observed in the vimentin content of T24 cells (**Figure 4A** and **Supplementary Figure S3A**). However, expression of vimentin was clearly detected in BFTC909 cells along with obvious polymerized morphology (**Figure 4B** and **Supplementary Figure S3B**). The quantitative results showed that the vimentin expression after exposure to immune cells was high in J82 and low in T24 (**Figure 4B**).

We then examined whether the vimentin responded differently within sensitive and resistant lung cancer cells upon attack by NK cells. After co-culture of the lung cells H292 and A549 with NK cells for 6 h, no change in the vimentin expression was observed in H292 cells (**Figure 4C** and **Supplementary Figure S3C**). Conversely, an expansion of vimentin network was detected in A549 cells (**Figure 4C** and **Supplementary Figure S3D**). Therefore, we found that vimentin was upregulated in highly immunoresistant cancer cells in response to immune cell attack (**Figure 4D**).

**Figure 5:**
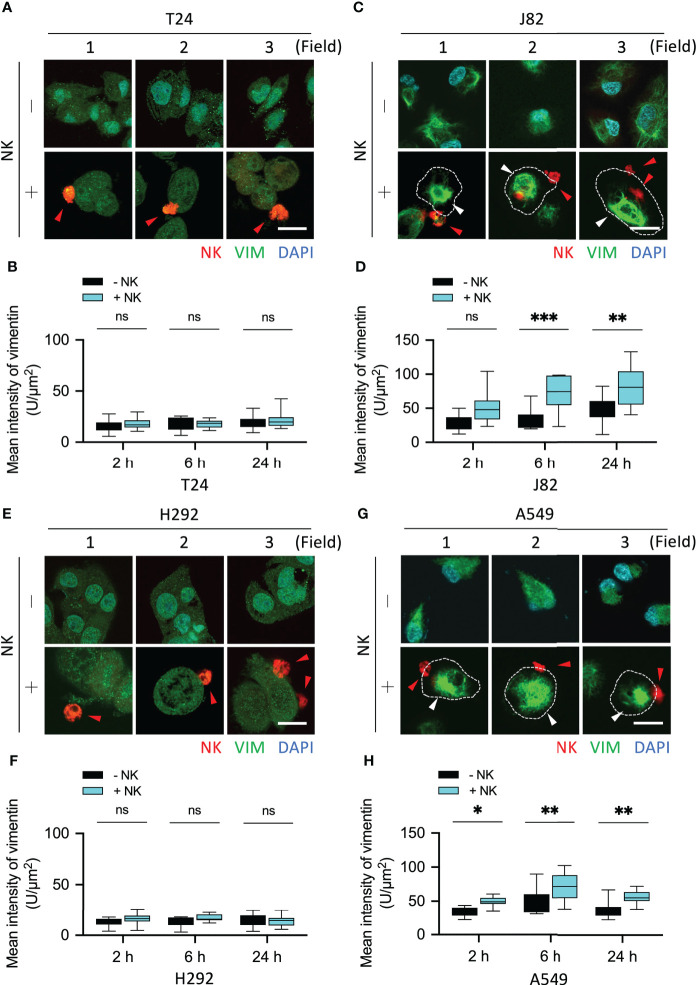
NK cells induced vimentin expression in the immune-resistant cancer cells. **(A, C)** Comparison of the expression of vimentin in NK cell-interacted cancer cells. T24 and J82 cells were incubated with NK cells for 2, 6 and 24 h, and the expression of vimentin was detected using immunofluorescence assays. **(B, D)** The intensity of vimentin was measured and quantified by ImageJ. **(E, G)** H292 and A549 cells were incubated with NK cells for 2, 6 and 24 h, followed by the detection of vimentin expression. **(F, H)** Quantification of the intensity of vimentin using ImageJ. ns, not significant; *P < 0.05; **P < 0.01; ***P < 0.001. Data are presented as mean ± s.d. (two-way ANOVA with Sidak's multiple comparisons test). Red arrowhead, NK cell; white arrowhead, vimentin. Scale bars, 20 µm. Data derived from 10 fields, each including 2–3 cells.

The mean fluorescence intensity of vimentin in T24 cells co-cultured with NK cells for 2, 6, and 24 h was NK-:NK+ = 16.02:18.39, 17.6:17.76, and 19.4:21.78, respectively (all P = ns) (**Figures 5A, B**). The mean fluorescence intensity of vimentin in J82 cells after 2, 6, and 24 h with NK cell co-culture was NK-:NK+ = 28.85:50.09, 33.68:71.39, and 46.48:91.96, respectively (P = ns, < 0.001, and < 0.01, respectively) (**Figures 5C, D**). In J82 cancer cells, the mean intensity of vimentin was higher at 24 h than at 2 h after adding NK cells (2 and 24 h; 50.09 and 81.96, respectively, *P* < 0.01) (**Figure 5D**).

The mean fluorescence intensity of vimentin in H292 cells co-cultured with NK cells for 2, 6, and 24 h was NK-:NK+ = 12.3:15.66, 12.87:16.8, and 14.74:13.7, respectively (all P = ns) (**Figures 5E, F**). The mean fluorescence intensity of vimentin in A549 cells after 2, 6, and 24 h with NK cells co-culture was NK-:NK+ = 33.46:49.28, 49.75:70.64, and 37.16:55.85, respectively (P < 0.05, < 0.01, and < 0.01, respectively) (**Figures 5G, H**). In summary, the accumulation of actin filaments and the upregulation of vimentin were involved in the response of cancer cells to immune cells. However, the presence of such phenomena in UTUC tumors is what we want to explore next.

### Invasion of NK cells in UTUC upregulated vimentin in cancer cells

First, we investigated whether changes in vimentin of cancer cells caused by NK cells could be detected in cancer patients. Staining of immune cells and vimentin was performed in stage III tumors of UTUC. Tumors were fixed and cut into slices for immunofluorescence and immunohistochemistry staining. The NK1.1 is expressed primary on NK cells and also found on NKT cells, a subset of CD4^+^ T cells and dendritic cells. The specificity of NK1.1 and vimentin antibodies was verified using an IgG antibody (**Supplementary Figure S4**). In the cold areas for the immune responses, i.e., the tumor without invasion by NK cells, less vimentin signal was detected either.

**Figure 6:**
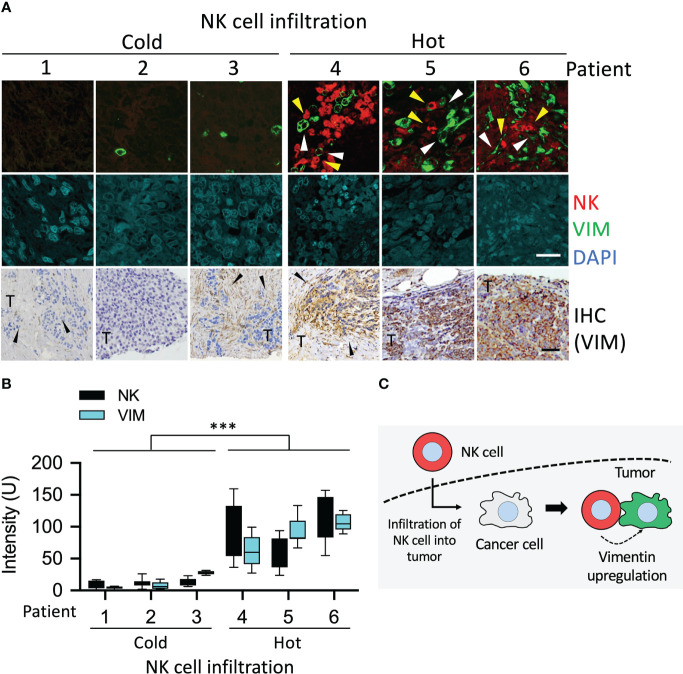
Upregulation of vimentin in NK cell infiltrated tumor of UTUC. **(A)** Different levels of NK cell-infiltrated tumors from patients with UTUC were collected. The tissue sections of UTUC were co-stained with NK1.1 and vimentin antibodies followed by detection of fluorescence signals using a confocal fluorescence microscope. Vimentin was also detected using immunohistochemistry staining. **(B)** The fluorescence intensity of NK1.1 and upregulated vimentin in the tumor was quantified by ImageJ. ***P < 0.001. Data are presented as means ± s.d. (two-way ANOVA) from three fields (n = 3). **(C)** Schematic represented the upregulation of vimentin induced by NK cells. Yellow arrowhead, NK cell; white arrowhead, vimentin. White scale bars, 50 µm, Black scale bars, 100 µm.

Interestingly, in tumor islands invaded by a small number of NK cells, vimentin was significantly detectable in the cancer cells surrounding the NK cells (**Figure 6A**). This was more apparent in tumors invaded by a large number of NK cells (hot areas), wherein strong vimentin signals were detected in the cancer cells around the NK cells (**Figure 6A**). Vimentin was also detected by immunohistochemistry assay, and hematoxylin staining was used to visualize cell morphology of the tissue (**Figure 6A**). Quantification of these results showed a high level of correlation between the NK cells in tumor and vimentin in cancer cells (**Figure 6B**). In tumors where no NK cell invasion was detected, the vimentin signal was extremely low (mean intensity 7.1 – 20.9), while in tumor islands with high level of invasion by NK cells, the mean intensity of vimentin was 79.1 – 113.7 (**Figure 6B**). These results showed that vimentin expression in the cancer cells caused by invasion of NK cells was indeed upregulated in UTUC tumors (**Figure 6C**). In summary, the expression of vimentin was stimulated in tumors invaded by NK cells.

### Interference with actin filament polymerization promoted the cytotoxicity of NK cells

**Figure 7:**
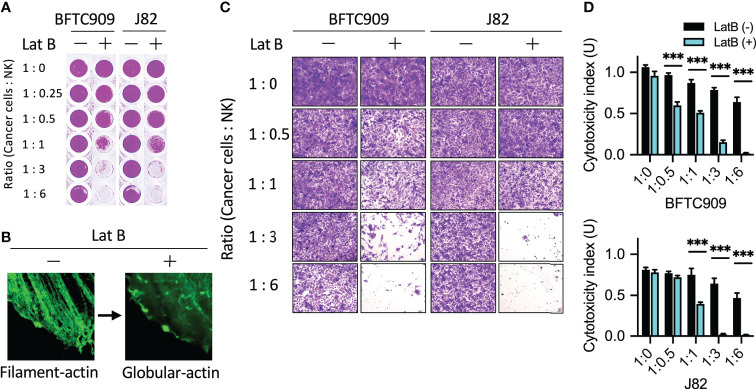
Disruption of actin filaments formation enhanced NK cell-mediated cell death.** (A)** Pretreatment of latrunculin B, an actin filaments inhibitor, increased NK cytotoxicity using urothelial cancer cells. Cells were seeded in the 96-well plate and pretreated with 0.5 µM latrunculin B for 6 h following which the inhibitor was removed. The cancer cells were then incubated with NK cells for 24 h. **(B)** Representative images of latrunculin B showing reduced actin filaments formation. **(C)** J82 and BFTC909 cells were co-cultured with NK cells for 24 h and stained with crystal violet. The stained cells were observed using a microscope. **(D)** The stained cells were counted and statistically analysis was performed using the Prism software. ***P < 0.001. Data are presented as means ± s.d. (two-way ANOVA with Tukey’s multiple comparisons test) and experiments were repeated three times (n = 3). Latrunculin B, LatB.

Reorganization of actin cytoskeleton plays a role in promoting the migration and metastasis of malignant tumors. Our previous study reported that inhibiting actin reorganization limited tumor progression and metastasis (30). The results in **Figures 1** and **2** demonstrated that NK cells promoted the accumulation of actin filaments in resistant cancer cells. Therefore, we further investigated whether actin filament polymerization affected the cytotoxicity of immune cells (**Figure 7A**). Immunoresistant cells (BFTC909 and A549) were pretreated with an actin polymerization inhibitor (latrunculin B), which could inhibit actin polymerization (**Figure 7B**). After pretreatment with latrunculin B for 6 h, the inhibitor was removed, and NK cells were added for another 24 h (the ratios of cancer cells to NK cells were 1:0.25; 1:0.5, 1:1, 1:3, and 1:6). Subsequently, cells were fixed and stained with crystal violet dye (**Figures 7C, D**), and the morphology after treatment with NK cells was observed under a microscope. The result demonstrated that when BFTC909 cells were treated at a ratio of 1:6, the morphology of the cells was normal. By contrast, after the NK cells were co-incubated with cancer cells pre-treated with latrunculin B at a ratio of 1:3 for 24 h, the cancer cells changed shape from round to elongated type. We speculated that pretreatment with latrunculin B altered the regulation of cancer cell cytoskeletal proteins to respond to NK cell attack (**Figure 7C**). The results revealed that BFTC909 cells were not sensitive to NK cells before treatment with latrunculin B, with 39.7% of cell death at a ratio to NK cells of 1:6. In contrast, cells became sensitive to NK cells after treatment with latrunculin B, with approximately 84.6% of cells dying after co-culture with NK cells at a ratio of 1:3. Up to 47.2% of cells died after co-culture with NK cells a ratio of 1:1 for 24 h (**Figure 7D**).

Similar results were obtained using another resistant cancer cell line, J82. Statistical quantification of the cells showed that the rate of cell death was 43.2% at a ratio to NK cells of 1:6, while the rate reached > 90% in cells treated with latrunculin B after co-culture with NK cells a ratio of 1:3 for 24 h (**Figure 7C**). This result showed that in urothelial cancer cells, pretreatment with actin polymerization inhibitors changed the response of BFTC909 and J82 cells to NK cells from resistant to sensitive cells. Our results show that the inhibition of actin reorganization in resistant cancer cells may enhance immune cell attack.

### Silencing vimentin promoted the cytotoxicity of NK cells

**Figure 8:**
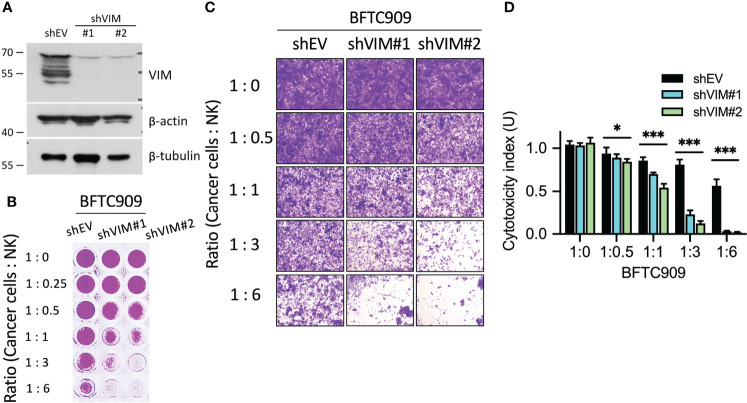
Silencing of vimentin enhanced NK cell-mediated cell death. **(A)** Silencing efficacy of shVIM#1 and shVIM#2 by western blot. **(B)** Silencing of vimentin in urothelial cancer cells increased NK cytotoxicity. BFTC909 cells were silenced with shEV, shVIM#1 or shVIM#2 and stable cells were screened. The stable cells were seeded in the 96-well plate 24 h and then incubated with NK cells for 24 h. **(C)** BFTC909-shEV, shVIM#1 or shVIM#2 cells were co-cultured with NK cells for 24 h and stained with crystal violet. The stained cells were observed using a microscope. **(D)** The stained cells were counted and statistically analysis was performed using the Prism software. *P < 0.05; ***P < 0.001. Data are presented as means ± s.d. (two-way ANOVA with Tukey’s multiple comparisons test) and experiments were repeated three times (n = 3).

Previously, the vimentin expression was found to be increased in many cancers, such as colorectal and lung cancer. However, the correlation between vimentin and tumor progression remained controversial (14). As shown in **Figures 2** and **3**, high vimentin expression was found when cancer cells were attacked by NK cells. Such phenomena were also verified in patient tumor tissues (**Figures 6A, B**). Based on this, we further explored if vimentin expression affected the cytotoxicity of immune cells. The experiment was designed to suppress the expression of vimentin in the immunoresistant cells (BFTC909) by using shRNA, which inhibited the expression of vimentin (**Figure 8A**). A stable cell line was selected from cells with knocked-down vimentin and was then co-cultured with NK cells for 24 h (the ratios of cancer cells to NK cells were 1:0.25, 1:0.5, 1:1, 1:3, and 1:6). Cells were then fixed and stained with crystal violet dye (**Figure 8B**). The morphology of cancer cells treated with NK cells was observed using a microscope, and shrinkage of some cells was observed in BFTC909 cells at a ratio of 1:6, while other cells showed normal morphology. After silencing vimentin, cancer cells were co-cultured with NK cells at a ratio of 1:3 for 24 h and the cancer cells were sensitive to immune cells (**Figure 8C**). Statistical quantification of the cells found that BFTC909 cells were not sensitive to NK cells, with 46.1% of dead cells observed at an NK cell ratio of 1:6. In contrast, after silencing vimentin, BFTC909 cells became sensitive to NK cells, and 77.9% - 89% of cells died after co-incubation with NK cells at a ratio of 1:3 for 24 h. After co-culture with NK cells at a ratio of 1:1 for 24 h, 32.1% – 49.1% of the cells were dead (**Figure 8D**). These results demonstrated that silencing vimentin in urothelial cancer cells transformed the response of BFTC909 cells to NK cells from being resistant to being sensitive. It is implied from this conclusion that inhibiting the expression of vimentin in resistant cancer cells may enhance the cytotoxicity of immune cells.

## Discussion

Tumor evasion of immune surveillance is the main reason for the inefficiency of immunotherapy. Here, we demonstrated that actin cytoskeleton reorganization and vimentin expression in urothelial cancer cells were resistant to NK cell-induced cytotoxicity. Using the NK cytotoxicity assay, we found that in urothelial and lung cancer cell lines, two types of cells can be distinguished, which differ in their sensitivity and resistance to NK cell attack (**Figure 1**). Notably, when resistant cancer cells were attacked by NK cells, obvious actin filaments aggregation occurred at the immune synapse, whereas this phenomenon was not evident in sensitive cells (**Figure 2**). The same phenomenon was observed when detecting vimentin expression, and resistant cells exhibited vimentin upregulation after NK cell attack (**Figure 3**). Tissue staining provided direct evidence that tumors with high NK cell infiltration exhibited more vimentin expression (**Figure 4**). Furthermore, in highly resistant cells, pretreatment with latrunculin B promoted the cytotoxicity of NK cells (**Figure 5**). Inhibition of vimentin expression by shRNA was also sufficient to convert the resistant cancer cell lines to a sensitive phenotype (**Figure 6**). Taken together, these findings demonstrated that actin reorganization and vimentin expression of cancer cells affected NK cell cytotoxicity.

NK cell cytotoxicity is regulated by a multistep process. NK cells interact with intercellular adhesion molecule 1 (ICAM1) and activating receptors, such as NCR, NKG2D, and DNAM1 *via* CD2, selectin CD62L, and adhesion integrin receptors (LFA1 and Mac1) (31, 32). These processes cooperate with integrin signaling to promote the formation of NK cell immunological synapses (NKIS). The formation of adhesion ring junctions after NK cell contact with cancer cells requires signaling mediated by cytoskeleton remodeling (33). F-actin accumulates in NKIS and is important for promoting the aggregation of various activating receptors. F-actin organization is a major driver of immune synapse formation and lipid raft polarization between NK and target cells (34–36). Actin retrograde flow (ARF) regulates NK cell signaling and NK cell activation and inhibition. This mechanotransduction process is regulated by the dynamics of actomyosin (37–39).

The stiffness of the matrix affected the cytotoxicity of NK cells and promoted the secretion of the NK cytokine interferon-gamma (IFN-γ). Cytokine release increases when NK cells interact with stiffer substrates. Cell stiffness changes continuously during the tumor process, with primary tumor cells being stiffer than healthy cells, and highly metastatic cells being less stiff (40). Additionally, viral infections can increase stiffness by inducing cortical actin rearrangement (41). Therefore, we speculated that the stiffness of the cells could affect the responsiveness of NK cells to infiltrate tumors.

Al Absi et al. found that actin cytoskeleton remodeling drives cancer cells to escape NK cell-mediated cytotoxicity (19). For example, after breast cancer cells encounter NK cells, actin accumulates at the immune synapses, which in turn limits the accumulation of the cytotoxic molecule granzyme B (GrzB) in cancer cells. Thus, cancer cells are protected from lysis and apoptosis (42, 43). The urothelial and lung cancer cell lines used in this study also exhibited actin accumulation at the immune synapses in resistant cancer cells but not in NK-sensitive cancer cells (**Figure 2**). This suggests that sensitive cancer cells may have a higher capacity to accumulate actin filaments at the immune synapses than resistant cancer cells. This reveals that differences in the accumulation of actin filaments in cancer cells at immune synapses may affect NK cell killing and cancer cell resistance. Most studies so far have focused on actin reorganization regulating NK cell function, and our study showed that interference with actin reorganization and vimentin expression affected the ability of NK cells to kill cancer cells (**Figures 2**, **3**). Our findings reveal the molecular and biophysical mechanisms by which dynamic cytoskeletal networks within cancer cells regulate NK cytotoxicity. The expression of vimentin, which coordinates lytic granule trafficking, MTOC polarization and actin dynamics at the immune synapses, remains unclear. An in-depth exploration of this information will help classify cancer cell sensitivity to NK cells and provide opportunities for future cancer treatment.

Al Absi et al. demonstrated that actin accumulation significantly protects cancer cells from NK-mediated cytotoxicity (19). A possible reason why cancer cells escape death is the creation of a physical barrier that renders lytic particles ineffective. Another escape mechanism can be the aggregation of the inhibitory checkpoint ligands. We speculated that actin reorganization and vimentin expression may induce PD-L1 coalescence at NKIS to evade NK cell surveillance. Studies from Al Absi et al. also showed that the inhibition of actin nucleation factors, such as N-WASP or Cdc42, in cancer cells restored NK cell-mediated cytotoxicity (19). In our study, interference with the remodeling of actin cytoskeleton and inhibition of vimentin can effectively promote NK cytotoxicity. Therefore, approaches aiming to modulate cytoskeletal proteins of cancer cells may have therapeutic potential.

Periodic exposure to the tumor microenvironment may induce NK cell exhaustion (44–48). Such exhausted NK cells typically express one or more inhibitory checkpoint receptors, such as programmed cell death protein 1 (PD-1), TIM-3, or TIGIT, which limits their cellular activity. Exhausted NK cells show reduced proliferation, cytokine release and degranulation. Actin cytoskeleton remodeling of target cells affects CTL recognition and lysis efficiency of NK cells. Changes in the actin dynamics in cancer cells result in immune evasion by inhibiting immune synapse formation or interfering with effector functions (19, 49–51). Decreased actin on the surface of cancer cell membranes may not only prevent strong adhesion of immune cells but also reduce the strength of mechanoreceptor signaling, resulting in insufficient NK activation signaling at the immune synapse. Enhanced actin dynamics in cancer cells reduce the cytotoxic enzymes perforin and GrzB (19). The actin cytoskeleton flanking the immune synapse determines lymphocyte cytotoxic attack and cancer cell resistance.

An interesting finding of our study was that urothelial cancer cell lines contain two types of cells, one of which was immunoresistant cells that responded to NK cell attack by regulating actin reorganization and vimentin expression while surviving this attack. Another group of immune-sensitive cells lacked this regulatory function and was highly sensitive to lysis by NK cells. Therefore, when studying cytotoxic immune responses against tumors, the immunoresistance caused by reorganization of cytoskeletal proteins in response to immune cell attack should be considered. We propose that targeting cytoskeleton remodeling in cancer cells may improve the efficacy of immunotherapy.

## Data availability statement

The original contributions presented in the study are included in the article/**Supplementary Material**. Further inquiries can be directed to the corresponding authors.

## Ethics statement

The studies involving human participants were reviewed and approved by Institutional Review Committee of Chang-Gung Medical Foundation (IRB number: 201504731B0 on 14 September 2015; 202101713B0 on 4 October 2021). The patients/participants provided their written informed consent to participate in this study.

## Author contributions

Conceived and designed the experiments: J-MP, H-YL, Y-LC, and H-LL. Performed the experiments: J-MP, J-WL, and Y-TW. Contributed reagents/materials/analysis tools: C-FC, J-HC, and H-LL. Wrote the manuscript: J-MP, J-WL. All authors contributed to the article and approved the submitted version.

## Funding

The work was supported by research grants from Chang Gung Memorial Hospital (Grant number: CMRPG8M0221, CMRPG8L0611, CMRPG8L0721, CMRPG8K1091 and CMRPG8K1092) and Ministry of Science and Technology (Grant number: MOST110-2320-B-182A-017), Taiwan. We are grateful to the National RNAi Core of Taiwan for the lentivirus-based shRNA clones.

## Conflict of interest

The authors declare that the research was conducted in the absence of any commercial or financial relationships that could be construed as a potential conflict of interest.

## Publisher’s note

All claims expressed in this article are solely those of the authors and do not necessarily represent those of their affiliated organizations, or those of the publisher, the editors and the reviewers. Any product that may be evaluated in this article, or claim that may be made by its manufacturer, is not guaranteed or endorsed by the publisher.
