# Supplementary material for: Evasion of NK cell immune surveillance via the vimentin-mediated cytoskeleton remodeling
Source: Front Immunol. 2022 Aug 11;13:883178. doi: 10.3389/fimmu.2022.883178 (PMC9402923; doi:10.3389/fimmu.2022.883178)

Supplementary Figure 1. Quantitative analysis of the actin response at immune synapses. (A) Front regions of the immune synapse bound to NK cells are marked with dotted rectangles (F), and unbound regions at the rear are marked with dotted rectangles (R). Mean fluorescence intensity (unit/μm^2) = F-R in the immune synapse, showing the level of F-actin aggregation. The fluorescence intensity of actin response at immune synapses was measured and quantified by ImageJ. (B) Representative images of quantification of the actin response at immune synapse are shown. Red arrowhead, NK cell; White arrowhead, actin filaments. Scale bars, 20 μm.

Supplementary Figure 2. NK cell induced actin response at immune synapses in cancer cells. (A-D) Comparison of the actin response in cancer cells after the addition of NK cells. T24, J82, H292 or A549 cells were incubated with NK cells for 6 h, and the expression of actin filaments was detected using an immunofluorescence assay. Red arrowhead, NK cell; white arrowhead, actin filaments. Scale bars, 50 μm. Magnified views are shown on the right.

Supplementary Figure 3. NK cell induced vimentin expression in cancer cells. (A-D) Comparison of the vimentin expression in cancer cells after the addition of NK cells. T24, J82, H292 or A549 cells were incubated with NK cells for 6 h, and the expression of vimentin was detected using an immunofluorescence assay. Red arrowhead, NK cell; white arrowhead, actin filaments. Scale bars, 50 μm. Magnified views are shown on the right.

Supplementary Figure 4. Evaluation of the specificity of NK and vimentin antibodies used for immunofluorescence assays. (A) Detection of NK cells in tumor tissues from patients with UTUC. The tissue sections of UTUC were stained with IgG or NK1.1 antibodies and secondary antibodies conjugated with Alexa Fluro 594 followed by detection of fluorescence signals using a confocal fluorescence microscope. (B) The tissue sections of UTUC were stained with IgG or vimentin antibodies and secondary antibodies conjugated with Alexa Fluro 488. Yellow arrowhead, NK cells; white arrowhead, vimentin. Scale bars, 100 μm.

Supplementary Figure 1

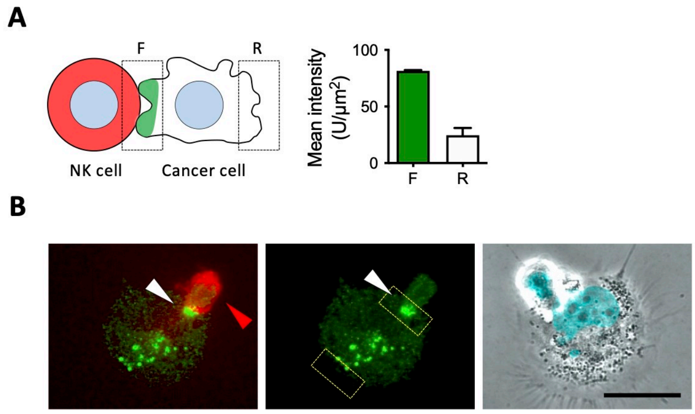

Supplementary Figure 2

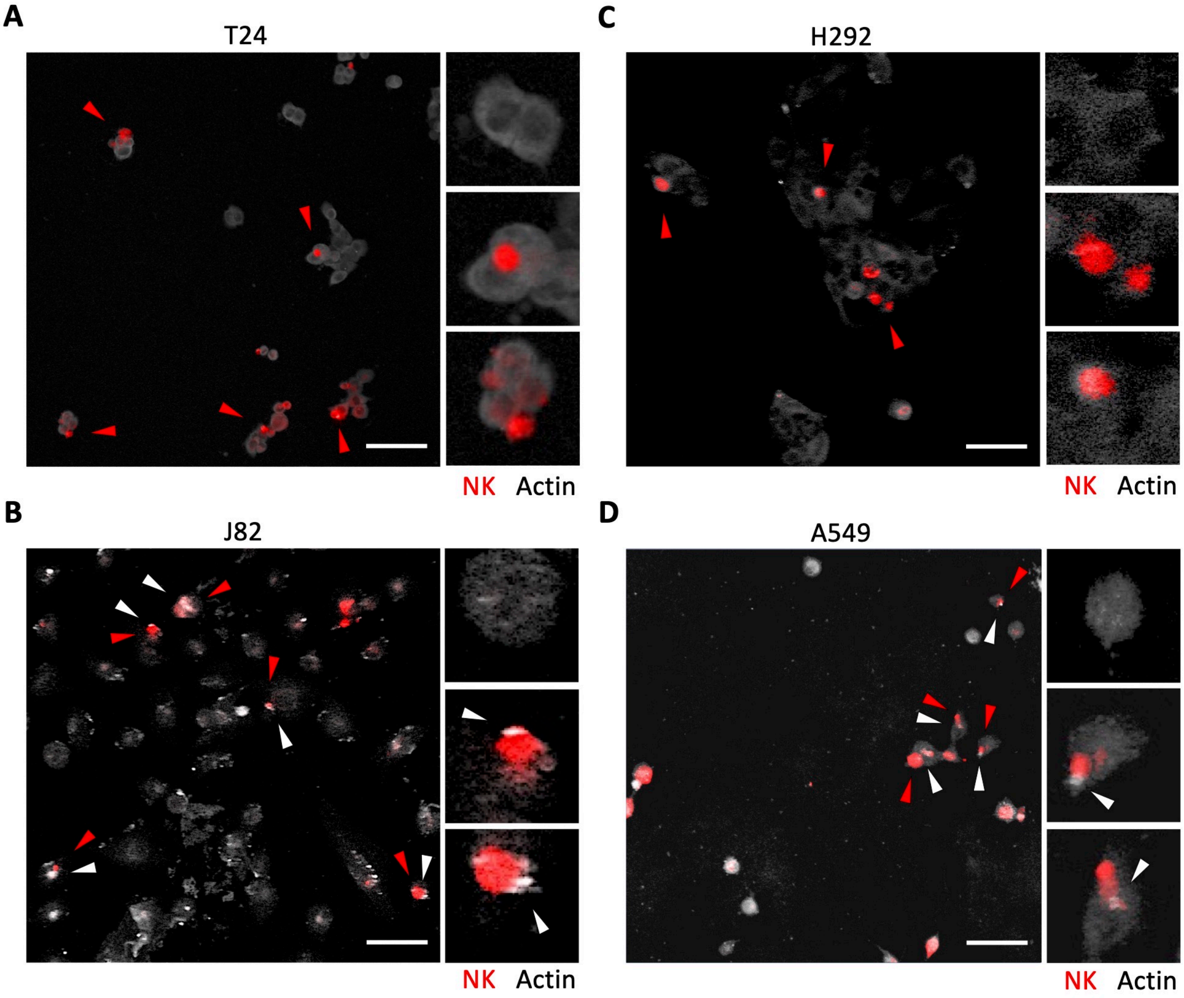

Supplementary Figure 3

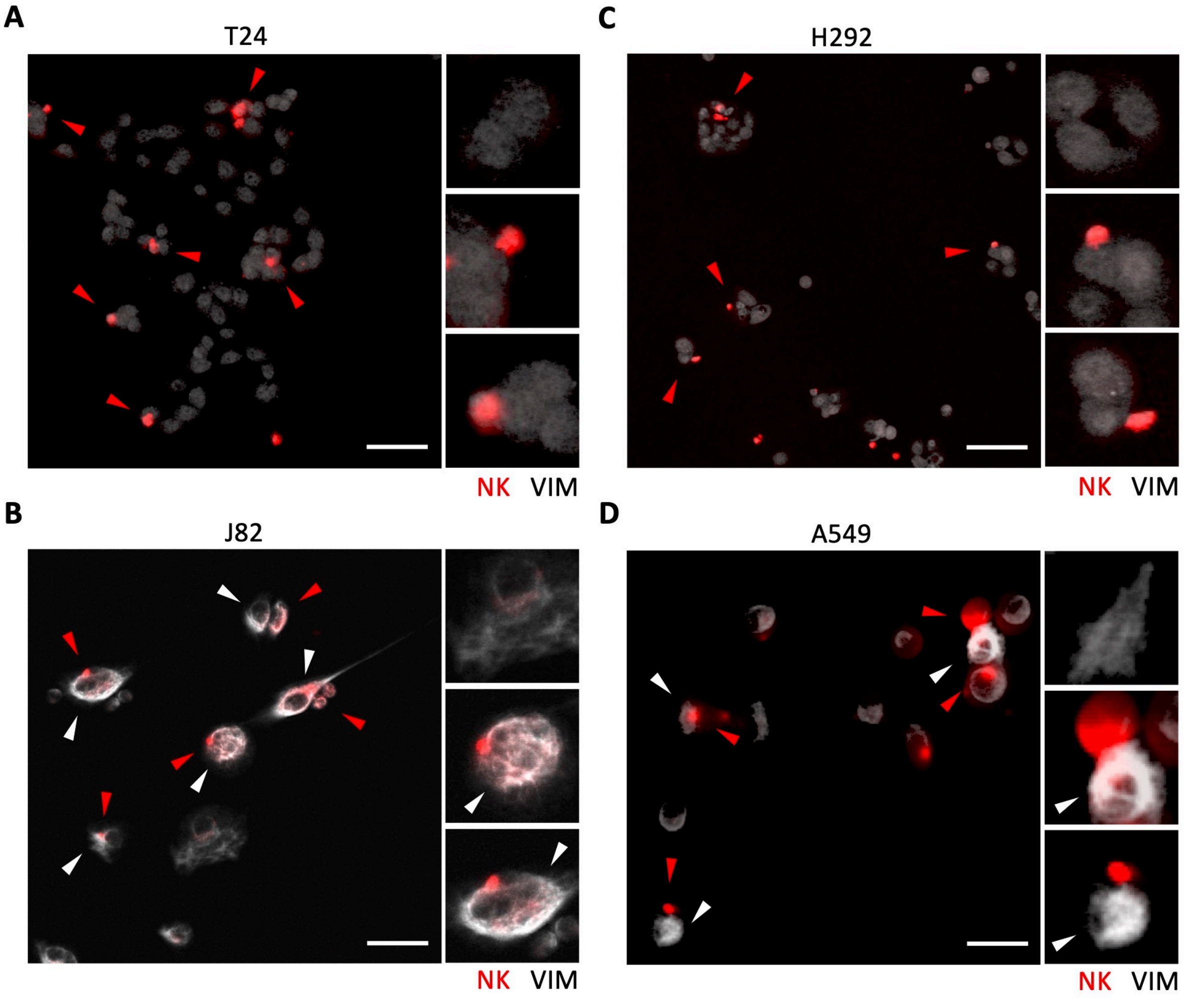

Supplementary Figure 4

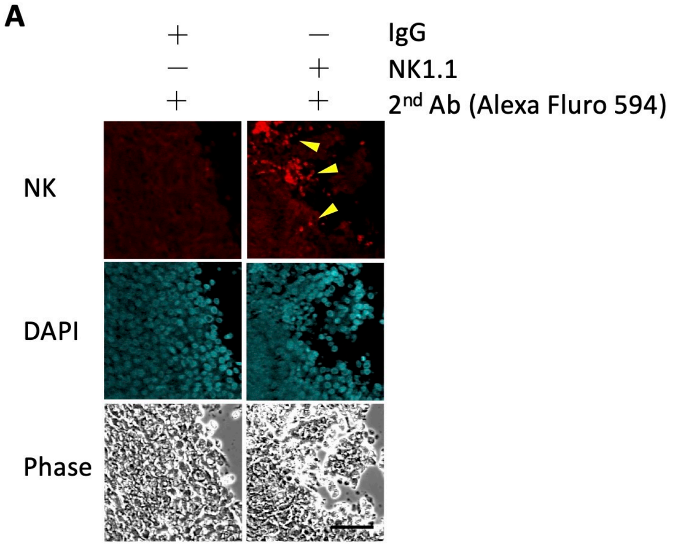

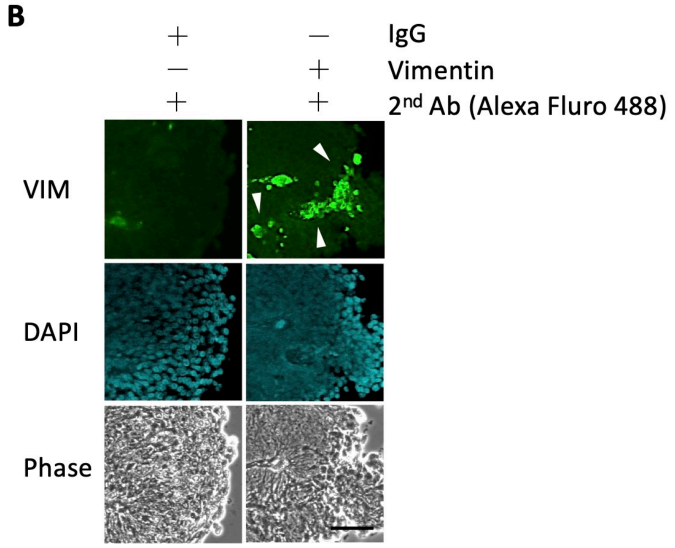

Supplement: Supplementary file 1 [file DataSheet_1.pdf]
